# Clinical Scoring Systems in the Newborn Calf: An Overview

**DOI:** 10.3390/ani12213013

**Published:** 2022-11-03

**Authors:** Monica Probo, Maria Cristina Veronesi

**Affiliations:** Department of Veterinary Medicine and Animal Sciences, Università degli Studi di Milano, Via dell’università, 6-26900 Lodi, Italy

**Keywords:** clinical score, newborn calf, APGAR, viability, diarrhea, respiratory disease

## Abstract

**Simple Summary:**

Clinical scores are gaining increasing popularity in veterinary medicine thanks to their multiple advantages, which include quickness, ease, and convenience of use. This review discusses the applications of some already-known clinical scores in newborn calf management at birth and during the first weeks of age. Clinical scores are employed to assess newborn calf viability and to diagnose and monitor neonatal calf diarrhea and respiratory diseases, helping the clinician promptly recognize calves needing medical assistance. This review discusses limitations pertaining to their use and encourages efforts towards a greater consistency in definition and validation.

**Abstract:**

A scoring system is an instrument that enables the scorers, including farmers, technicians, and veterinarians, to adopt a systematic approach for diagnosis or monitoring, as it decreases bias and confounding and increases objectivity. Practically, it is a number assigned to a patient that correlates with a probability that a diagnosis can be confirmed or that a specific outcome will follow. This article examines the clinical scores designed or adapted to bovine medicine that aim to assess newborn calf viability and to diagnose and monitor neonatal calf diarrhea and respiratory diseases, helping the clinician promptly recognize calves needing medical assistance. Despite the large number of clinical scores described in the literature, these are still barely used in farm animal practice; possibly, the complexity of the scores and missing recommendations for intervention are reasons for their lack of popularity as well as the crosswise lack of consistency among scores designed for the same purpose. Further research is needed in this regard to increase scores validation and encourage their application in bovine calf neonatology.

## 1. Introduction

Calf mortality rates reported from European countries vary from 3.87% [1] to 7% [2], and the highest risk of death occurs during the first 4 weeks of life [2]. Calf mortality during this period can be distinguished between perinatal mortality (stillborn after 270 d of gestation—24 h after birth) and neonatal mortality (death between 1 and 28 d of age) [3]. Dystocia has been reported as the most important cause of perinatal mortality [4], together with anoxia and trauma following dystocia and, to a much lesser extent, intrauterine death and premature placental expulsion [5], while pneumonia and diarrhea are the most common causes of neonatal death [2,6,7]. However, in most cases, the cause of calf mortality is multifactorial, often due to a combination of dam factors, infective agents, and nonoptimal management.

Although definitions and age categories chosen for calf mortality investigation are not always comparable, a recent review [7] estimated that the incidence risk of perinatal mortality increased by an absolute 0.02 between 1990 and 2000. Increased perinatal mortality in modern dairy countries lowers production efficiency and delays genetic progress by reducing availability of calves as replacements for voluntary culling or for sale [8] and additionally incurs welfare costs. Consequently, control programs that minimize mortality in neonatal calves are worthful to farmers.

A prompt identification of poor health newborn calves is essential for the rapid and efficient intervention, and it is hence a crucial objective in the modern dairy industry. A valuable tool in this purpose is represented by scoring systems, which enable the scorers (farmers, technicians, and veterinarians) to adopt a systematic approach in this regard [9]. Scoring systems are means to objectively evaluate and express a clinical condition and to stratify patients according to a specified outcome; they can be diagnosis-specific or diagnosis-independent [10]. Through clinical scores, the subjectivity of the operator is removed, and the data base is enlarged compared to those resulting from the experience of a single clinician [11,12]. When assessing the severity of illness and predicting an outcome, scores should not be applied alone but in combination with other determinants; moreover, predictions should be applied on a population basis rather than on individuals [10]. When selecting a clinical score for a specific intent, the following characteristics should be evaluated: validated predictive accuracy, transferability to the intended patient population, and ease of application. In the literature, several calf scoring systems are described for relatively similar purposes, and the lack of uniformity requires the knowledge of the pros and cons of each scoring system in order to appropriately choose the best one for a specific setting.

This review paper aims to describe and discuss the available scoring systems regarding newborn calf viability (Section 2), neonatal calf diarrhea (Section 3), and calf respiratory disease (Section 4).

## 2. Viability Scores

### 2.1. The APGAR Score

In developed countries, pre- and post-natal care programs have resulted in a very high success rate for prevention of problems in newborn babies. Part of this success is due to a standard requirement for the completion of a health and viability score within a few minutes after birth. The first-ever newborn viability score was developed for human babies by Virginia Apgar, M.D., with the main goal to allow the routine assessment of all newborns around the world and under all possible clinical conditions. The score consisted of the following five signs: hearth rate, respiratory effort, reflex irritability, muscle tone, and color, which aimed to assess for hemodynamic compromise such as cyanosis, hypoperfusion, bradycardia, hypotonia, respiratory depression, or apnea. Each sign was rated zero, one, or two, and the sum of scores given to each parameter allow to divide newborns into three categories: 0–3 points as severely depressed, 4–6 points as moderately depressed, and 7–10 points as excellent condition [13]. In 1962, it was proposed that Dr. Virginia Apgar’s surname could become itself an acronym for rapid memorization of the five parameters: A for appearance (skin color), P for pulse, G for grimace (reflex irritability), A for activity or attitude (muscle tone), and R for respiration. In human medicine, this score is still nowadays executed at 1 min after birth and repeated at 5 and 10 min. Strengths of the APGAR score include its feasibility under every condition, ease to be performed even by non-specialist personnel, low degree of instrumental employment, quick and feasible viability classification of newborns, and good short-term survival prognostic power [14]. The APGAR score was designed with the purpose of early detection of poor-viability newborns, and several studies have demonstrated its usefulness for this purpose. It is important to remark that the APGAR score was not created for the purpose of making long-term predictions about the newborn’s future health and growth.

Within the framework of veterinary medicine, APGAR scores have been developed for swine [15], horse [16,17], dog [18], cat [19,20], sheep [21], donkeys [22], and bovine species (see below). All these scores take inspiration from the original, and the majority are constituted by five signs rated zero, one, or two. In the choice of these parameters, it is important to consider their ease of detection and the absence of interference with maternal care and/or newborn resuscitation. Regarding bovine species, the parameters that are usually taken into account during APGAR score evaluation are the following:

- Heart rate (HR): HR is an indicator of overall vitality. Immediately after calving, a high HR of about 100–150 bpm is considered as physiologic, followed by a physiological decrease in the first hours. In at-risk calves, an initial tachycardia is observed (HR > 150 bpm), followed by bradycardia (HR < 80 bpm) [23,24].
- Respiration: Respiratory rate and respiratory rhythm are established indicators of overall vitality. Transient tachypnoea after birth is physiologic in the newborn calf. In at-risk calves, primary or secondary apnea and/or dyspnea can be observed [24].
- Mucous membranes color: It is considered a standard parameter in traditional APGAR scores related to blood oxygenation and peripheral perfusion, as cyanosis of buccal and lingual mucous membrane indicates prolonged dystocia and hypoxemia [24]. Homerosky et al. [25] reported that adoption of this parameter is more important for its correlation with hematocrit rather than with blood oxygenation.
- Grimace response: A multitude of tests have been proposed for assessment of reflex irritability. Mee [24] suggested to evaluate head shake by placing a finger or straw in nostrils, the corneal reflex by gently pressing eyelid, the suckling reflex by placing a finger in oral cavity, the tongue withdrawal by trying to pull the tongue, and the pedal reflex by pinching the interdigital space of any limb. Some authors [25,26] recommended to use suckling reflex (SR) alone or integrated with modified APGAR scores to assess the vitality of newborn calves. Typically, the calf has a frequency of ≥80 suckling movements/min. In according to [27,28], these tests are arguably the most correlated to blood pH. In fact, prolonged hypoxia causes a reduction in O_2_ cerebral levels and a subsequent central nervous system depression, which is responsible for impaired reflexes [29]. Moreover, [25] reported that SR and tongue withdrawal are extremely promising indicators of acidosis, especially for their correlation with blood parameters such as HCO_3_^−^, base excess (BE), and L-lactate. The suckling reflex is associated with acidosis subsequent to diarrhea and can be used to estimate BE [30]. Tongue withdrawal, evaluated as complete or incomplete, differs significantly based on blood L-lactate concentration [25].
- Muscle tone: This parameter is directly correlated along the response to reflexes stimulation [24,27,28]. Muscle tone can be indirectly evaluated through expression of neonatal behaviors such as head-rightening and attempt to stand [31].
- Rectal temperature: Normally, after parturition, calves have a higher temperature (39–40 °C) than the physiological one, which returns normal within three to five hours. Vermorel et al. [32] observed hyperthermia (>39.5 °C) followed by severe hypothermia (<37.0 °C) in calves born after prolonged and difficult calving; moreover, return of rectal temperature to normal values is slower in poor-viability calves.
- Hair coat appearance: Normally, calves are born covered by placental fluid. Meconium staining is observed following anal sphincter relaxation, a direct consequence of fetal stress derived from intrauterine hypoxia [33]. Moreover, a positive correlation between umbilical blood L-lactate concentration and meconium staining was demonstrated [34].

Additional parameters to be evaluated during the first hours after birth are:

- Time to attain sternal recumbency (T-SR) as indicators of newborn calf viability. T-SR is considered one of the best predictive parameters for newborn viability, as viable newborn calves attain sternal recumbence within few minutes. T-SR reflects the combined function of cardiovascular, locomotor, and nervous systems. Schuijt and Taverne [35] observed that T-SR was significantly longer in calves born after forcefully extraction.
- Attempts to rise and time for standing up: Viable calves attempt to stand within 15 min and reach the standing position within 1 h [36]. As for T-SR, these innate behaviors reflect a good functioning of cardiovascular, locomotor, and nervous systems. On the contrary, poor-viability calves either do not show or delay this innate behavior [24].

As emerges from the literature review, the first modified APGAR score for calves was developed by Mulling [37] using signs of asphyxia for scoring. Later on, researchers found that this modified APGAR score was only marginally correlated with the results of blood gas analysis [38], and they concluded that Mulling’s APGAR score was not indicated for the correct assessment of newborn calf viability, as it did not appropriately classify calves into viability groups based on acid–base status.

In 1981, Born proposed to evaluate four parameters and to score each from 0 to 3. The sum of these scores were interpreted as follows: 0–3, poor viability; 4–6, depressed; 7–10, good viability. The following year, Szenci [27] proposed a score system for evaluation of viability based on muscle tone and cardiac status, in which the latter parameter was considered only in problematic cases. Significant correlations were found between newborn tonicity and acid–base balance [27].

A first score that combined the parameters evidenced by [37] along with SR, T-SR, and other “behaviors” was proposed by Mee [24]. This score used visual and physical measures easily recordable in the field practice, both for farm staff and vets. Mee [24] suggested to assess the vigor of the calf immediately after calving by testing its reflexes and by the time to head-righting, to achieve sternal recumbence, to attempt to stand, and to stand (temporal limits for good vigor were 3, 5, 20, and 60 min, respectively). If the calf exhibits superficial abdominal breathing or has weak reflexes, or if it takes more than 15 min to achieve SR, the prognosis is poor. It is important to remark that these parameters are all “key indicators” of the perinatal etho-physiological profile and are correlated with newborn calf acid–base balance [24,25]. More recently, Lange and coauthors [39] have proposed a modified APGAR score system, with calves scored 0–3 points rated as depressed, those scored 4–6 as endangered for death, and those scored 7–10 as vital.

### 2.2. The VIGOR Score

Good vigor is a vital characteristic of newborn calves because it promotes behavior fundamental for survival, such as achieving SR, standing, finding the udder, and colostrum intake [31]. Adoption of parameters proposed by Mee [24] led to creation of a different score than the modified APGAR score. This score was developed at the University of Guelph (Guelph-ON-Canada), and it was called Calf VIGOR Score [40]. In this case, the word VIGOR is also an acronym: V—visual appearance; I—initiation of movement; G—general responsiveness; O—oxygenation; and R—rates. The final score is composed by 10 parameters subdivided in the five aforementioned VIGOR categories. These parameters are:

- V—meconium staining, tongue/head;
- I—calf movement;
- G—head shake in response to straw in nasal cavity, tongue pinch, eye reflex;
- O—mucous membrane color, length of tongue;
- R—heart rate, respiration.

Each parameter is rated 0, 1, 2, or 3 except for eye reflex, length of tongue, HR, and respiration, which are rated 0, 1, and 2. Contrary to previous scores, a score of 0 corresponds to normal, and increasing points indicate decreasing responsiveness. The sum of individual scores gives a result that can vary from 0 to 26, and based on final score, the authors proposed a subdivision of calves into five groups: 0–1 = excellent vitality, 2–4 = very good vitality, 5–6 = good vitality, 7–10 marginal vitality, and ≥11 poor vitality. To facilitate measurement of newborn calf vitality, the University of Wisconsin (Madison, WI, USA) developed an application for practical in-field employment (https://www.vetmed.wisc.edu/dms/fapm/apps/cvs.htm accessed on 24 August 2022).

### 2.3. Criticism and Limitations

As shown above, plenty of modified scores have been used by researchers in attempt to classify the vitality of calves; however, these index-based scores have substantially failed to be adopted by cattle producers and veterinarians outside research settings [25]. Possibly, the complexity of the scores and missing recommendations for intervention are reasons for their lack of popularity as well as the inability to consistently identify compromised calves. Moreover, differently from small animals, implementation of blood samples for investigations on acid–base balances and other variables in large animal newborns is practicable without special efforts, thus promoting instrumental viability assessment in this species. Homerosky et al. [25] suggested to combine a subset of APGAR parameters with easily attainable calving characteristics, with the aim of developing an evidence-based assessment tool. Certainly, this tool can facilitate prompt identification of acidemic newborn calves by producers and allows rapid veterinary intervention. Compared to the already well-validated employment of portable blood gas analyzers, an appropriate score would be cheaper and easier to perform, as it does not require blood sampling or even an expensive equipment. Moreover, it may be useful to identify those calves that survive immediately after calving but are at risk for reduced pre-weaning health and performances. Although there have been several attempts to establish a score to assess newborn calf vitality, further research is needed in this regard, as a practical tool that can be used to assess newborn calf vitality on farms with ease and accuracy has not yet been validated.

## 3. Neonatal Calf Diarrhea Score

Neonatal calf diarrhea (NCD) is one of the most common multifactorial diseases that cattle practitioners and farmers must deal with. Published data about the incidence and importance of this disease vary by country, type of productive system, year, and other factors. The incidence of the disease observed by different researchers ranges from the lowest levels in Sweden and Denmark (9.8 and 10.3%) to an average of 24–30% in Europe and USA [6,41,42]. The mortality of calves suffering from diarrhea ranges between 15–30% in Europe [3] and exceeds 50% in the United States [43].

The negative impact of neonatal diarrhea derives not only from its direct consequences (mortality and treatments) but also from its effects on herd profitability. Animals overcoming diarrhea show long-term effects on growth and on susceptibility to other diseases, such as respiratory illness [6,44]. Adequate management of rearing pens and calf care is essential since it can minimize morbidity and mortality of neonatal diarrhea outbreaks [45].

Neonatal calf diarrhea is usually accompanied by dehydration, acidemia, and electrolyte imbalance [46,47,48,49]. Blood gas analysis is widely regarded as the gold-standard test to assess the severity of metabolic acidosis, strong ion difference, and electrolyte derangements in diarrheic calves [50,51,52]. Nevertheless, performing laboratory tests on the farm is difficult, and clinical examination remains the first inescapable step for detecting and scoring the neonatal calf diarrhea at preliminary stages. Several studies have highlighted the strong correlation between clinical signs and the blood gas variables pH, bicarbonate (HCO_3_^−^), and base excess (BE) [48,49,53,54,55]. The ideal scoring system for neonatal calf diarrhea is the one that best correlates with blood parameters and that can be easily used also by non-trained primary caregivers.

A common practice surrounding diarrhea is the use of fecal consistency to identify the occurrence and degree of diarrhea [56]. This scoring was first mentioned by Larson et al. [57], who suggested a score based on fluidity, where a score of 1 is considered normal (firm but not hard, original form is distorted slightly after dropping to floor and settling), 2 is soft (does not hold form, piles but spreads slightly), 3 is runny (spreads readily to about 6 mm depth), and 4 is watery (liquid consistency, splatters). Kertz and Chester-Jones [58] further cemented this four-level scoring in a review suggesting that fecal consistency scoring is needed to ensure proper reporting in calf experimental data. McGuirk [56], however, suggested using a slightly modified 0 to 3 scale and classified diarrhea as a score of 2 (loose but enough consistency to remain on bedding) or 3 (watery feces that sift through the bedding).

Regardless of the scoring system, the majority of studies evaluating diarrhea in dairy calves use a fecal consistency scoring system. To objectivate this measure, Bellosa et al. [59] evaluated the accuracy of fecal consistency scoring as a mean to predict percent of fecal dry matter (DM), finding that the higher the fecal consistency score, the lower the percent DM of the feces. Recently, Renaud et al. [60] showed that fecal consistency scoring can predict a decline in fecal DM and thus that a higher numerical fecal consistency score may be an indicator of increased diarrhea severity. Fecal scoring is commonly used by producers for clinical decision making, such as when to provide fluid therapy [56,61], and it has merit as a reliable assessment of fluid loss (dehydration) associated with diarrhea [60]. In 1998, Constable and coauthors [47] investigated the use of potentially useful clinical factors to evaluate hydration status (extent of enophthalmos; skin-tent duration on neck, thorax, and upper and lower eyelids; heart rate; mean central venous pressure; peripheral and core temperatures; core–peripheral and rectal–extremity temperature difference) and concluded that the best predictors of degree of dehydration were the extent of enophthalmos and skin elasticity on neck and thorax.

All the above-mentioned scoring systems may allow for early detection of diarrhea and thus early intervention, which would mitigate severity of dehydration and, consequently, of metabolic acidosis. Nevertheless, they remain incomplete indicator systems when treatment has to be established, particularly referring to the correction of acidosis and electrolyte imbalance. Subjective methods of quantifying the degree of acidosis in NCD would in fact be of specific importance to obviate the need for portable blood gas analyzers. Some authors [46,62] have tried to develop a clinical score for estimating bicarbonate replacement requirements in NCD, echoed by [53] and more recently by [55]. Nakagawa and coauthors [53] proposed a depression score to evaluate acid–base status based on dehydration (enophthalmos), neurologic (suckle reflex, ability to stand, menace reflex, tactile responses), and cardiovascular (warmth of extremities and oral cavity) signs, according to previous guidelines [63]. These variable scores were summed to yield a minimum score of 0 in healthy calves and a possible maximum score of 15 in severely affected calves. This score appeared to be negatively correlated with blood values of pH, BE, HCO_3_^−^, and TCO_2_, and a cut-off value score was identified at 6.5 to differentiate severe from mild metabolic acidosis. Moreover, Boccardo and coauthors [64] described the score applied to NCD patients to clinically estimate dehydration and acidosis as a result of the combination of vigor score, dehydration score, and suckle reflex score; this score was then used as criteria for allocating calves to different therapeutic protocols. Sayers et al. [55] investigated the predictive capability of a five-point clinical assessment scoring (CAS) chart to differentiate severity of NCD; this CAS included calf demeanor, ear position, mobility, suckle reflex, enophthalmos, and desire-to-feed variables. Results indicated that blood pH, HCO_3_^−^, and BE were strongly and significantly correlated with CAS, but this was not intended to act as a validated scoring tool to inform the timing of intervention or treatment of diarrheic calves. In fact, the presented CAS well differentiated both clinically normal and advanced cases from the other severity classes but had reduced accuracy in differentiating mild and moderate cases. Essentially, CAS would be useful in diagnosing NCD in field, avoiding the underestimation of doubtful cases.

### Criticism and Limitations

Further enhancements to improve severity classification for this clinical scoring is required, also considering other possible clinical variables to be measured that may lead to a more standardized approach with reduced bias. Nevertheless, use of scores as a component of the overall clinical assessment of the individual is appropriate, whereas use of the score as the sole means of justifying a treatment or euthanasia decision is not. As first approach, caregivers should be trained how to correctly identify those calves needing assistance. Literature review clearly shows how only the combination of different clinical scores (fecal, dehydration, vigor) can provide an accurate snapshot of the situation. With this aim, a combination of a fecal score [56,57,59] and a clinical score similar to those proposed by [55,64] would enable caregivers to differentiate mild NCD cases, in which a tailored treatment protocol can be enough, from more severe NCD cases, where skilled veterinarian assistance and complementary tools are mandatory for proper addressment and treatment. This would also allow to restrict the employment of complementary and more expensive investigations to the most severe cases.

## 4. Respiratory Diseases Score

Whether around the time of birth or later, respiratory problems of calves are, together with calf scour, the first cause of death during the neonatal period and the most important diseases in calves older than 30 days [45]. In the USA, respiratory disease is responsible for 21.3% of mortality in pre-weaned dairy calves and 50.4% in weaned calves [43]. In addition to treatment and labor costs, other negative sequelae from respiratory diseases include increased mortality and culling as well as poor growth and reproduction [6,41,65]. Early diagnosis may improve the chances of treatment success [66] and help avoid unjustified and inappropriate use of antimicrobials. The reference standard for diagnosing bovine respiratory disease (BRD) is necropsy in combination with histopathological, microbiological, molecular, and biochemical testing methods [67]. In the live animal, thoracic ultrasound and radiography can be used to diagnose BRD but require expensive equipment and operator training [68,69].

A major advantage of clinical scoring systems is that they do not require expensive equipment or diagnostics. The idea of applying clinical scoring systems to BRD diagnosis is not new: the first BRD score was published in 1977 as a mean to classify the severity of BRD in calves experimentally infected with BRSV or BVDV [70]. This score was developed as a part of a research that aimed at thoroughness and completeness; for that purpose, it accounted for 17 predictors beyond hematologic data, and it is not suitable for field work.

In 2014, other authors [71] suggested three options of scoring systems to assess the BRD in pre-weaned dairy calves. These systems differed for the number of clinical parameters that were taken into account (six in the first and third method; seven in the second) and for the score appointed to each parameter (1 point or 2 points). In the end, the minimum total score to reach for diagnosing BRD in each system also differed (4, ≥4, or ≥5 for first, second, and third system, respectively). Sensitivity was 95.4%, 89.3% and 89.4% for the first, second and third system, respectively, while specificity was 88.6%, 92.8% and 90.8% for the first, second and third system, respectively. Each of the proposed systems therefore offers different accuracy levels for the on-farm diagnosis of BRD in dairy calves, but no one of them aims at representing the criterion for choosing among different therapeutic approaches.

In the same year, McGuirk and Peek [72] proposed a scoring system based on four clinical signs in order to promptly identify calves that should be treated for BRD [72]. A respiratory disease score was calculated based on rectal temperature, the character of nasal discharge, eye or ear appearance, and presence of a cough. This respiratory disease score developed by researchers at Wisconsin University (WI score) is the sum of points from the four categories of clinical signs, with increasing values representing progressive severity. Points range from 0 to 3 as clinical signs progress from normal (0) to mildly abnormal (1), to moderately abnormal (2), and to severely abnormal (3). Calves with a total respiratory score ≥5 or that have two or more clinical parameters with score 2 or 3 are considered to have respiratory disease. A recent study has estimated the sensitivity and specificity of the WI scoring systems at 55.4% and 58.0%, respectively [73].

In 2019, Maier and coauthors [74] tested the application of a score based on the variables cough (2 points), abnormal respiration (1 point), low body condition (5 points), sunken eyes (4 points), and a 24 h ambient temperature range > 15 °C (1 point) with a 2-point cutoff for a BRD suspect score; the score was tested on weaned calves and showed a 77.0% screening sensitivity, 100% diagnostic sensitivity, and 61.9% specificity. An alternative model did not contain a score for the covariate 24 h ambient temperature range and had a 1-point cutoff. The alternative model had a screening sensitivity of 84.2%, diagnostic sensitivity of 100%, and specificity of 45.7%. Adding rectal temperature ≥ 39.2 °C (102.5 °F) as a second-tier test increased specificity and lowered the screening sensitivity and diagnostic sensitivity in both models.

### Criticism and Limitations

In light of the great number of score systems proposed for BRD diagnosis, it is clear that the choice of the appropriate score must go through the evaluation of available resources and final goals. Besides helping with single-case diagnosis, the respiratory scoring system should become one of the farm tools employed for calf health-screening programs. Among the cited scores, the WI score is the one requiring the evaluation of the least number of clinical signs and is therefore the most suitable for field work. Nevertheless, the WI score subdivides each of its clinical signs into four levels, and inexperienced operators may encounter difficulty in appropriately classifying clinical signs in calves without overlapping. A two-level approach may therefore be applied within the herd health-screening program; as a first step, a respiratory score easy to calculate, such as the one proposed by [74], would help identifying ill calves that should be thereafter evaluated through a more accurate clinical score. The WI score may represent this “second step” score since the evaluation of the rectal temperature requires handling the single calf alone. When manipulation of the single calf is necessary, clinical scores may be matched with the focused lung ultrasonography proposed by [75] in order to ameliorate score definition and improve the accuracy of the diagnosis.

## 5. Conclusions

Many diagnosis-specific and diagnosis-independent veterinary scores have been presented in recent years. The clinical scoring systems reduce subjectivity and increase timeliness for diagnosis thanks to objective and easy criteria. Despite the increasing interest in this regard, clinical scores are still barely used in farm-animal practice; more research efforts are clearly needed, as clinical score spreading runs through an accepted and trusted development methodology and requires a validation on large number of patients. Score adoption in farm-animals medicine may therefore be maximized by the development of well-validated scores, which, however, do maintain the most essential features of quickness and ease of application. In particular, an agreement among the many different viability scores should be reached, as already seen in many other species, in order to also standardize the strategies of intervention. Regarding NCD, only the combination of different clinical scores (fecal, dehydration, vigor) can provide an accurate diagnosis and staging of severity of the disease, leading to a complementary investigation only when actually needed. Finally, for BRD, research efforts should be focused on combining clinical scores with focused lung ultrasonography in order to improve score sensitivity and specificity.
